# Distinct immune responses and virus shedding in pigs following aerosol, intra-nasal and contact infection with pandemic swine influenza A virus, A(H1N1)09

**DOI:** 10.1186/s13567-016-0390-5

**Published:** 2016-10-20

**Authors:** Johanneke D. Hemmink, Sophie B. Morgan, Mario Aramouni, Helen Everett, Francisco J. Salguero, Laetitia Canini, Emily Porter, Margo Chase-Topping, Katy Beck, Ronan Mac Loughlin, B. Veronica Carr, Ian H. Brown, Mick Bailey, Mark Woolhouse, Sharon M. Brookes, Bryan Charleston, Elma Tchilian

**Affiliations:** 1 The Pirbright Institute, Pirbright, UK; 2 Virology Department, Animal and Plant Health Agency, Weybridge, Addlestone, UK; 3 School of Veterinary Medicine, University of Surrey, Guilford, UK; 4 School of Veterinary Sciences, University of Bristol, Langford, UK; 5 Aerogen IDA Business park Dangan, Galway, Ireland; 6 Centre for Immunity, Infection and Evolution, University of Edinburgh, Kings Buildings, Edinburgh, UK; 7 Jenner Institute, University of Oxford, Oxford, UK

## Abstract

Influenza virus infection in pigs is a major farming problem, causing considerable economic loss and posing a zoonotic threat. In addition the pig is an excellent model for understanding immunity to influenza viruses as this is a natural host pathogen system. Experimentally, influenza virus is delivered to pigs intra-nasally, by intra-tracheal instillation or by aerosol, but there is little data comparing the outcome of different methods. We evaluated the shedding pattern, cytokine responses in nasal swabs and immune responses following delivery of low or high dose swine influenza pdmH1N1 virus to the respiratory tract of pigs intra-nasally or by aerosol and compared them to those induced in naturally infected contact pigs. Our data shows that natural infection by contact induces remarkably high innate and adaptive immune response, although the animals were exposed to a very low virus dose. In contacts, the kinetics of virus shedding were slow and prolonged and more similar to the low dose directly infected animals. In contrast the cytokine profile in nasal swabs, antibody and cellular immune responses of contacts more closely resemble immune responses in high dose directly inoculated animals. Consideration of these differences is important for studies of disease pathogenesis and assessment of vaccine protective efficacy.

**Electronic supplementary material:**

The online version of this article (doi:10.1186/s13567-016-0390-5) contains supplementary material, which is available to authorized users.

## Introduction

Influenza A virus (IAV) is an important zoonotic pathogen that can cause substantial mortality and rapidly disseminate through economically important avian (ducks and chickens) and mammalian (human, swine and other) populations [1–3]. H1N1 and H3N2 subtypes of IAV are endemic in pigs and humans, in addition to H1N2 in pigs. Because human origin viruses or viruses containing human origin gene segments frequently adapt to transmit efficiently in pigs [4, 5] the pig is a source of new viruses capable of initiating epidemics or pandemics in humans of mixed swine, human and avian origin [6].

As both pigs and humans are readily infected with IAVs of similar subtype, the pig is a robust and appropriate model for investigating both swine and human disease. Like humans, pigs are outbred, and physiologically, anatomically and immunologically similar to humans. The porcine lung also resembles the human in terms of its tracheobronchial tree structure, lung physiology, morphology and distribution of receptors bound by influenza A viruses [3, 7]. Thus studies of the infection dynamics of pandemic (pdm) A/(H1N1)09 origin viruses in pigs may also throw light on factors affecting transmission and infection in humans.

However very few studies have evaluated the importance of dose and route of delivery of swine influenza virus (SwIV) in experimental challenge studies. Experimentally SwIV is typically delivered to the airways of pigs by intra-nasal inoculation with a syringe [8, 9], intra-nasally with a mucosal atomisation device (MAD) [10–12] or by intra-tracheal instillation [13–17]. The intra-tracheal route is reported to result in infections that cause more severe morbidity [13, 14, 18, 19] that are a reflection of the greater virus replication in the lung, while severe morbidity is rare with intra-nasal challenge. Intra-tracheal delivery is widely used because of its reproducibility and consistency, however the virus is delivered to the lower respiratory tract (LRT) and bypasses the upper respiratory tract (URT), which is the natural route of infection. In contrast the aerosol route approximates more closely the natural route of transmission as it targets the LRT but via the URT [20, 21] and has been described as showing more severe clinical signs [22]. However, very few challenges have been performed using aerosol delivery. In order to determine the most relevant model for assessment of IAV pathogenesis, transmission, vaccine efficacy or therapeutic intervention, we examined whether experimental delivery of SwIV to the URT or LRT intra-nasally or by aerosol respectively, best represents natural infection. To do this we evaluated the virus shedding patterns and immune responses of pigs after intra-nasal (IN) and aerosol (AERO) challenge with different doses and compared these to animals that had become infected by contact transmission, the “natural” route.

## Materials and methods

### Animals and influenza virus challenge

Animal experiments were approved by the Pirbright Institute and APHA ethics committees, according to the UK Animal (Scientific Procedures) Act 1986. Eight to nine week old landrace cross, female pigs were obtained from a commercial high health status herd. All pigs used were derived from the same cohort, sourced at the same time and acclimatized for a period of 7 days. Pigs were screened for absence of IAV infection by matrix (M) gene real time RT-PCR [23] and antibody-free status was confirmed using haemagglutination inhibition (HAI) with 4 SwIV antigens. Pigs were inoculated with the strain A/Sw/Eng/1353/09, provided by Dr Sharon Brookes, APHA (DEFRA SwIV surveillance programme SW3401). The inoculum stock was propagated in the allantoic cavities of 9–11-day-old embryonated specific-pathogen-free hens’ eggs. For all serological and immunological assays, the virus was propagated in Madin-Darby canine kidney (MDCK) cells.

Groups of four pigs were challenged with either 1 × 10^4^ egg infectious doses EID_50_ (low dose) or groups of three pigs were challenged with 1 × 10^7^ EID_50_ (high) of virus. Virus delivery was performed either by aerosol (AERO) in 1 mL following sedation or by the intra-nasal route (IN) using a mucosal atomization device, MAD300 (Wolfe Tory Medical) with 2 mL of virus administered to each nostril, of non-sedated animals. For aerosol challenge an InnoSpire Deluxe Philips Respironics nebulizer was fixed to a small-sized anaesthetic mask held over the animal’s nose and mouth. The animals were sedated with 5 mg/kg ketamine hydrochloride and 1 mg/kg Stresnil: azaperone administered intramuscularly.

**Figure 1:**
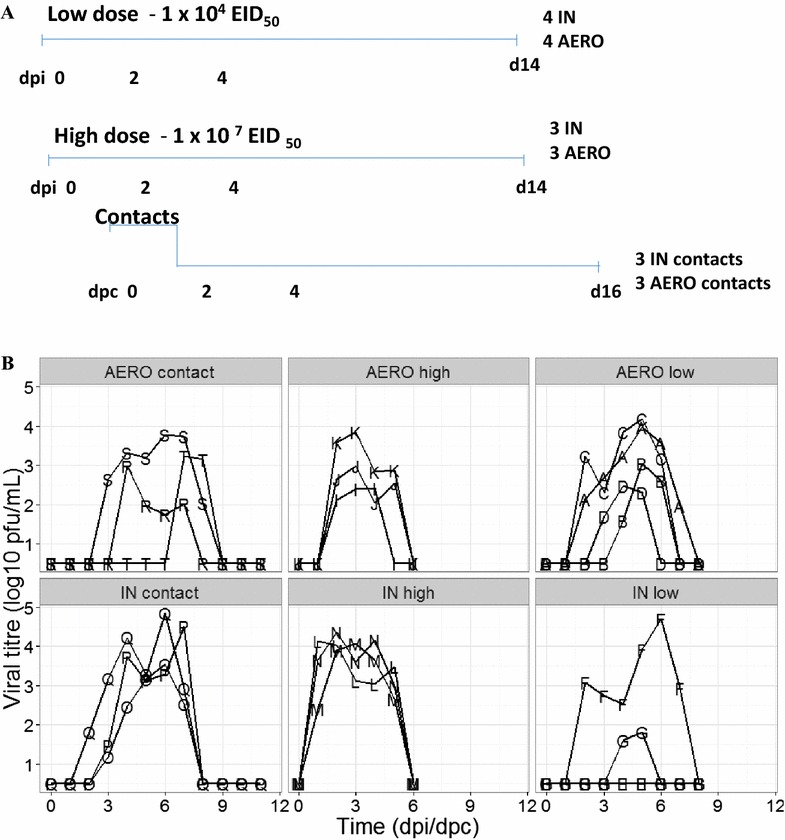
**Experimental design and virus shedding of AERO, intranasal and contact challenge. A** Pigs were challenged with 1 × 10^4^ EID_50_ (low) or 1 × 10^7^ EID_50_ (high) of A/Sw/Eng/1353/09 virus by aerosol (AERO) or intra-nasally (IN). After 2 days three naïve pigs were co housed with each of the AERO high or IN high animals and after further 2 days moved to separate rooms. Horizontal lines indicate the time scale of the experiment and the vertical bars show challenge, time of contact and sacrifice. **B** Viral titers in nasal swab suspensions for indicated groups. The viral titres are represented by markers. Each line represents an individual within the indicated group.

Two days after the high dose challenge, three naïve pigs were co-housed with the 3 AERO challenged pigs and three further naïve pigs with the 3 IN infected pigs for 48 h, after which they were moved to separate clean rooms (experimental design in Figure 1A).

In total 8 pigs were challenged with low dose: 4 by AERO (A, B, C, D) and 4 by IN (F, G, H, I); 6 were challenged with high dose: 3 by AERO (E, J, K) and 3 by IN (L, M, N); and 6 were infected were co-housed with infected pigs: 3 co-housed with AERO (R, S, T) challenged pigs and 3 with IN (O, P, Q) challenged.

Animals were monitored by observing demeanour, appetite and respiratory signs such as coughing and sneezing. Body temperatures were monitored using an Identichip Biothermal microchip. Animals were euthanized at 14 days post-infection (dpi) or 16 days post-contact (dpc) with an overdose of intravenous pentobarbital sodium.

### Aerosol droplet size characterization

The average droplet size produced by each of the MAD (IN) and nebuliser (AERO) devices was characterised using laser diffraction as previously described [24]. Briefly, the devices were loaded with PBS to their specified maximum fill volumes and connected to the inlet of the droplet sizer. The device under test was turned on and run until the entire dose was delivered. Testing was carried out in triplicate.

### Tissue sample processing

Four nasal swabs (two per nostril) were taken daily from day 0 until the day of euthanasia at the end of the study. The four nasal swabs from each sampling were placed together into 2 mL of Leibovitz medium (Gibco), containing 1% foetal bovine serum (FBS) and 1% penicillin/streptomycin. The tubes containing the swabs were agitated, swabs were removed, and the supernatant was aliquoted and stored at −80 °C for subsequent analysis.

Clotted and heparin anticoagulated blood samples were taken before the challenge and at 4, 7, 11, 14 dpi for the animals infected with a low dose of virus and at 3, 7, 10 and 14 dpi for animals infected with a high dose of virus, blood was taken from contact animals at 2, 5, 9, 12 and 16 dpc. For PBMC isolation heparinised blood was diluted 1:1 in PBS before density gradient centrifugation at 1200×*g* for 30 min over Histopaque^®^ 1.083 g/mL (Sigma-Aldrich). PBMC were harvested from the interface, washed in PBS and contaminating red blood cells lysed using ammonium chloride lysis buffer, washed again and cryopreserved in FBS (Gibco) with 10% (v/v) DMSO (Sigma).

Broncho-alveolar lavage (BAL) was performed *post mortem*, by washing the lung with 250 mL of PBS and harvesting 100 mL of the fluid. BAL cells were isolated by centrifuging the lavage fluid at 800 × *g* for 15 min, supernatant was aliquoted and frozen for antibody detection, the cell pellet was washed in PBS, filtered twice using a 70 µM cell strainer and cryopreserved. Tracheo-bronchial lymph nodes (TBLN) were dissected at *post mortem*, cut into 1–5 mm pieces, before further dissociation into a single cell suspension using the GentleMACS Octo (Miltenyi), and C tubes (Miltenyi) with 3 mL of complete medium RPMI (Gibco) supplemented with 10% FBS and penicillin/streptomycin. The cell suspension was filtered using a 70 μM cell strainer and washed in PBS. All cells were cryopreserved in FBS with 10% DMSO at a minimum concentration of 1 × 10^7^ cells/mL.

### Virus titration in nasal swabs

Viral titres in nasal swab suspensions were determined by plaque assay on MDCK cells. Duplicate samples were tenfold serially diluted and 100 μL medium added to confluent MDCK cells in 12 well tissue culture plates. After 1 h, the plates were washed and overlayed with 2 mL 1:3 2% (w/v) agarose:medium. Plates were incubated at 37 °C for 48 h and plaques visualized by staining the monolayer with 0.1% (v/v) crystal violet.

Viral RNA levels in nasal swabs were also quantified by RRT-qPCR by amplification of the M gene. RNA was extracted from 140 μL swab suspension using the QIAamp viral RNA mini kit and Biorobot extraction (Qiagen) according to the manufacturer’s protocol. The assay conditions used were as previously described [25]. Viral RNA present per mL of nasal swab suspension was correlated to relative equivalent units (REU) of infectious virus per millilitre using a ten-fold dilution series of RNA purified from infective allantoic fluid and using an inter-run standard containing RNA equivalent to a known EID_50_ titre of A/sw/Eng/1353/09.

### Pathological examination of lungs

To assess the lung pathology groups of 4 pigs were also challenged with 1.5 × 10^5^ pfu/pig of intermediate dose of MDCK-grown A/Sw/Eng/1353/09 (equivalent to 6 × 10^6^ EID_50_) by the IN or AERO routes. The MDCK- and egg-grown viruses used in this study were sequenced and confirmed to be identical. The animals were humanely killed 4 dpc with an overdose of pentobarbital sodium anaesthetic. At *post mortem* the lungs were removed and digital photographs taken of the dorsal and ventral aspects. Macroscopic pathology scoring was performed blind using Nikon-NIS Br software to determine the proportion of the total surface area of the lung (dorsal and ventral aspects) affected by typical influenza-like gross lesions.

### HAI assay

Influenza virus specific antibody (Ab) titres in serum and BAL fluid were determined by HAI using standard protocols [11]. Briefly, H1N1 HAI antibody titres were determined using 0.5% chicken red blood cells and A/Sw/Eng/1353/09 live antigen at a concentration of 4 HA units/mL.

### IFN-γ ELISPOT

Frequencies of IFN-γ secreting cells in PBMC, BAL cells and TBLN cells were determined by ELISPOT using previously cryopreserved cells. MultiScreen™-HA ELISPOT plates (Merck Millipore), were coated with 0.5 μg/mL of anti-pig IFN-γ, clone P2G10 (BD Pharmingen) in carbonate buffer and incubated at 4 °C overnight. The plates were washed 5 times in PBS and blocked using 4% (w/v) milk powder in PBS for 2 h. After 5 washes in PBS, 5 × 10^5^ cells were seeded in triplicate wells and stimulated with either live MDCK-grown A/Sw/Eng/1353/09 (MOI = 5), medium control or 10 μg/mL Con A (Sigma-Aldrich). Plates were incubated for 40 h at 37 °C in a 5% CO_2_ incubator, followed by five washes with PBS, 0.05% Tween20 and addition of 0.25 μg/mL anti-pig biotinylated IFN-γ detection Ab, clone P2C11 (BD Pharmingen). Plates were incubated for 2 h at room temperature, washed 5 times and streptavidin alkaline phosphatase (Invitrogen) was added for a further 1 h at room temperature. Spots were visualised using alkaline phosphatase substrate kit (BioRad) and the reaction was stopped using tap water. Immunospots were counted using the AID ELISPOT reader (AID Autoimmun Diagnostika). Results were expressed as number of IFN-γ-producing cell per 10^6^ cells after subtraction of the average number of IFN-γ^+^ cells in medium control wells.

### Flow cytometry

Cryopreserved PBMC and cells from TBLN and BAL were thawed and stimulated for 12 h at 37 °C with live MDCK-grown virus strain A/Sw/Eng/1353/09 (MOI 6 for BAL and PBMC, MOI 0.6 for TBLN) or MDCK mock supernatant as control. GolgiPlug (BD Biosciences) was added according to the manufacturer’s instructions for a further 5 h before intracellular cytokine staining. Cells were stained for surface markers with CD3ε-PeCy5 PPT3 (AbCam), biotinylated CD4 clone MIL17 (in-house), with secondary streptavidin-APC (Southern Biotec), CD8α-FITC MIL12 (AbD Serotec) and Near-Infrared Fixable Live/Dead stain (Invitrogen). Cells were permeabilized using Cytofix Cytoperm (BD Biosciences) as per the manufacturer’s instructions before intracellular staining (ICS) with IFN-γ PE P2G10 (AbD Serotec). Samples were fixed in 1% paraformaldehyde before analysis using an LSR Fortessa instrument (BD Biosciences).

Data was analysed using FlowJo v10 (Treestar), fluorescence of control samples stained with one primary antibody omitted were used to set gates. Samples were batch gated on lymphocytes based on SSCA/FSCA, followed by single cells on SSCH/SSCA. Live CD3 positive cells were analysed for expression of CD4 and CD8α. Boolean gating was used to determine the levels of IFN-γ in CD8α high, CD4CD8α double positive and CD4 T cell subsets.

### Detection of cytokines

The presence of IL-1β, IL-4, IL-6, IL-8, IL-10, IL-12, IFN-α, IFN-γ and TNFα in nasal swabs was determined using ProcartaPlex Cytokine&Chemokine panel 1 (nineplex, Affymetrix, eBioscience) according to the manufacturer’s instructions. Results were quantified on a Luminex^®^ 200™ and cytokine and chemokine concentrations in the samples were determined using a standard curve for each cytokine (with lower limits of detection respectively 0.092, 0.072, 0.428, 1.420, 1.250, 1.463, 0.00, 1.245 and 2.272 pg/mL). All results were obtained in duplicate and represented as the mean.

### Distribution of Evans Blue in lungs and pathological analysis after IN or AERO administration

Evans Blue (Sigma-Aldrich), 5% solution in PBS was administered by aerosol using either nebuliser or intra-nasal MAD device as described for the influenza A virus challenge above. After 20 min pigs were euthanized with an overdose of intravenous administered pentobarbital sodium. A detailed *post mortem* investigation of the upper and lower respiratory tract was conducted.

### Statistical analysis

To analyse influenza viral kinetics we estimated the initial viral load, *V*_*0*_, which is the necessary viral load at inoculation to induce the subsequent viral kinetic, the ascending slope, *s*_*1*_, the time of the peak, *T*_*max*_ and the descending slope, *s*_*2*_, using the segmented linear regression package called “segmented 0.5–0.0” in R 3.1 [26]. Parameter fitting was performed by maximum-likelihood method, using the log-scaled viral loads. The data was truncated to keep virus-positive samples. In addition we included two samples where the viral titre is below the limit of quantification, one before and one after the shedding period. We tested the association of the individual parameters between the dose groups (low, high, contact) or route group (AERO, IN, contact) using pairwise permutational t test from the package “RVAideMemoire 0.9–55” in R 3.1.1. We used 1000 permutations and *p* value was adjusted for multiple testing with the Benjamini and Hochberg method [27]. In the case of pigs which did not have a virus-positive sample on any occasion after the challenge, we performed the analysis after their exclusion. From the individual estimated parameters, we also predicted for each pig the height of viral titre peak computed as: *V*_*max*_ = *V*_*0*_ + *s*_*1*_ *T_*max*_ and characterized the viral shedding period as the time during which viral titre is >1 log_10_ (log_10_ PFU/mL). The start of shedding period was computed as *T*_*start*_ = *(1*−*V*_*0*_ *)/s*_*1*_, the end of the shedding period as *T*_*end*_ = *(1*−*(V*_*max*_−*s*_*2*_ *))/s*_*2*_ and the duration of shedding period as *T*_*end*_−*T*_*start*_.

IL-6, IL-8 and IL-1β were used for statistical analysis as greater than half the samples assayed showed levels above the limit of quantification. The remaining cytokines: IFNα, IFNγ, IL-10, IL12p40, IL-4 and TNFα had greater than 78% of their data below the lower limit of quantification. Data for IL-6, IL-8 and IL-1β were transformed prior to analysis to correct for non-normality. IL-8 was log_10_ transformed and IL-6 and IL-1β were square root transformed. Data that were below the lower limit of quantification (LLOQ) were set to half LLOQ. Only data up to (and including) day 6 were included in order to standardise the analysis. Baseline samples were excluded as the time of sampling was not equivalent for all pigs. Principal components analysis (PCA) was run on the cytokine data matrix (20 pigs, 3 variables and 6 time points) using PC-ORD software version 6.03 (MJM software Design, Gleneden Beach, OR). PCA was used with a Euclidian distance measure after relativizing by standard deviates of the columns. The final set of components was determined using stopping procedures [28]. A second matrix was created including virus titre, day and challenge groups. Data within the second matrix was overlaid onto the final PCA to look for associations with cytokine levels. The strength of the correlation along principal component (PC) 1–2 for continuous variables was measured using Kendall’s τ nonparametric correlation coefficient. Significance of the τ correlation was determined using proc freq in SAS version 9.1.3 (SAS Institute Inc., Cary, NC). Significance of the categorical variables were assessed using Multi-response Permutation Procedures (MRPP) analysis, a nonparametric procedure for testing the hypothesis of no difference between two and more groups [29].

To describe the immune responses of each treatment group, the number of input variables was reduced using PCA (R statistical software version 3.3.0) to investigate underlaying principal components. Data from the HAI assay, IFN-γ ELISPOT and flow cytometry were included in the analysis, virus and cytokine data were excluded. Principal components 1–5 (PC1–PC5) were submitted for analysis using individual general linear models, followed by post hoc tests using Tukey adjusted-least significant means.

## Results

### Kinetics of virus shedding following aerosol, intranasal and contact infection with influenza virus

To determine the role of dose and route of infection on virus shedding and immune responses, pigs were infected with A/Sw/Eng/1353/09 influenza virus. They were challenged either with a low (1 × 10^4^ EID_50_) or high (1 × 10^7^ EID_50_) dose of virus either by aerosol (AERO) using a nebuliser, which generates small droplets believed to target the lower respiratory tract (LRT) or intra-nasally (IN) using a MAD, which produces large droplets believed to be mostly deposited in the upper respiratory tract (URT). Using laser diffraction, the average droplet size produced by the MAD device was measured as 105.7 ± 14.1 μm. The fine particle fraction (FPF) less than 5 μm, i.e. the aerosol fraction considered capable of deposition in the LRT, was 0%. The average droplet size produced by the nebuliser was 3.8 ± 0.1 μm with a FPF of 63.4%. Groups of naïve pigs were co-housed with each of the high dose directly infected groups at 2 dpi and moved to separate rooms after a further 48 h (Figure 1A). The clinical signs observed were mild and none of the pigs developed moderate or severe disease. Gross pathology was minimal in all animals at the time of sacrifice at 14 dpi or 16 dpc.

Virus was detected in the nasal swabs of all 4 animals in the AERO low group, with the start of shedding between 2 and 4 dpi (Figure 1B). However only 2 of the 4 animals in the IN low dose group shed any detectable virus. In contrast, all animals in the IN high dose group started shedding virus 1 dpi and virus was detected until 5 dpi, while the animals in the AERO high dose started shedding virus at 2 dpi and virus could only be detected for 3 or 4 days. Furthermore the animals in the IN high group not only started shedding virus earlier, but also shed more virus compared to the AERO high group (Figure 1B). Because all IN high animals shed more virus, all of the three IN contacts were infected and shed virus by 3 dpc. In contrast only 2 of the 3 AERO contacts shed virus at 2 and 4 dpc, while the third animal started shedding on 7 dpc, most likely a result of contact infection from one of its companions.

To analyse the kinetics of virus shedding we estimated the initial viral load, *V*_*0*_, the ascending slope, *s*_*1*_, the time of the peak, *T*_*max*_, the descending slope, *s*_*2*_, and the start and duration of viral shedding (Table 1). We also used the estimated parameters to predict the height of peak virus shedding (Additional file 1). The IN and AERO contacts were combined in one group for analysis as they represent natural infection (analysis with separate IN or AERO contacts gave similar results, not shown). The effects of either route or dose of challenge on viral kinetics were compared between the groups and with the contacts. Analysis by route showed that *V*_*0*_ was significantly different between the groups, with the IN high group showing the highest viral load, followed by the AERO high and contacts (Table 1). The viral load measured at 1 dpi in the high IN group should be considered with caution as it is not possible to differentiate virus shed from input virus. The end of the viral shedding period was also significantly different between contacts and the other groups as the contacts shed until 9.3 dpc (*T*_*end*_ ranging between 5.9 dpi for AERO high and 7.3 dpi for AERO low, Table 1). Analysis by dose on the other hand, indicates that the contacts are similar to the low dose pigs in all parameters except for *V*_*0*_ and the end of shedding while they showed multiple significant differences from high dose animals.

**Table 1:** **Median parameters describing influenza viral kinetics for the different challenge groups for virus titre determined by plaque assay**

Parameter	Unit	IN high	IN low	AERO high	AERO low	Cont.	*p* value for dose groups testing*			*p* value for route groups testing*		
		*N* = 3	*N* = 2^a^	*N* = 3	*N* = 4	*N* = 6	Cont. vs. high	Cont. vs. low	Low vs. high	Cont. vs. AERO	Cont. vs. IN	AERO vs. IN
V_0_	log_10_ PFU/mL	0.49 [0.48; 0.53]	0.41 [0.34; 0.47]	0.16 [0.15; 0.22]	0.21 [0.07; 0.33]	−0.02 [−0.05; 0.01]	0.024	0.024	0.711	0.038	0.009	0.009
s_1_	log_10_ PFU/d	3.34 [2.52; 3.52]	0.45 [0.34; 0.56]	1.05 [0.89; 1.18]	0.63 [0.50; 0.77]	0.63 [0.51; 0.69]	0.012	0.755	0.012	0.22	0.22	0.22
Tmax	d	1.42 [1.28; 2.00]	5.62 [5.31; 5.93]	3.16 [3.01; 3.25]	5.12 [4.62; 5.52]	5.60 [4.70; 5.72]	0.006	0.589	0.012	0.13	0.13	0.43
s_2_	log_10_ PFU/mL/d	−0.81 [−0.99; −0.73]	−1.41 [−1.92; −0.90]	−0.96 [−1.07; −0.83]	−1.21 [-1.41; −0.92]	−0.68 [−0.73; −0.63]	0.11	0.15	0.33	0.048	0.261	0.837
Predicted peak	log_10_ PFU/mL	4.97 [4.84; 5.09]	3.09 [2.25; 3.93]	3.18 [2.95; 3.72]	3.48 [2.49; 4.40]	3.45 [2.43; 3.96]	0.34	0.98	0.34	0.82	0.47	0.47
T_start_	dpi	0.16 [0.15; 0.21]	1.95 [1.31;2.58]	0.80 [0.73; 0.89]	1.33 [0.88; 1.87]	1.69 [1.42; 1.96]	0.018	0.579	0.054	0.32	0.32	0.66
T_end_	dpi	6.56 [6.27; 6.65]	6.92 [6.48; 7.36]	5.96 [5.56; 5.99]	7.24 [7.00; 7.39]	9.30 [9.11; 9.51]	0.012	0.012	0.024	0.012	0.012	0.831
Predicted dura tion of shedding period	d	6.40 [6.06; 6.50]	4.97 [3.90; 6.04]	5.17 [4.67; 5.26]	5.74 [4.86; 6.60]	7.52 [6.57; 8.10]	0.072	0.096	0.839	0.03	0.16	0.70

[Q1–Q3]: represents the interval between the first and third quartile; N: number of pigs; Median computed from the individual parameters.

^a^2 pigs do not have a virus-positive sample on any occasion after the challenge.

* *p* value computed from pairwise permtuational t test using 1000 permutation and with *p* value adjusted for multiple testing using Benjamini & Hochberg method. Cont. is for contact.

We also measured virus shedding by RRT-qPCR, which gave similar results although the sensitivity was higher, resulting in molecular detection of low equivalent titres of virus in comparison to plaque assay for infectious virions. Overall there was a good agreement between viral titre measured by plaque assay and by PCR (Additional file 2), although molecular detection enabled monitoring of virus shedding kinetics over a longer duration. By RRT-qPCR, only *T*_*max*_ was significantly different between groups (*p* = 0.01) with an earlier peak for pigs inoculated with high dose (2.3 dpi for IN high) than for pigs inoculated with low dose or by contact (5.1 dpc). This is consistent with other studies demonstrating that viral titre measured by plaque assay represent infectious virus, whereas PCR measure total infectious and non-infectious viral RNA [30].

Taken together these data indicate that a lower dose is required for AERO infection compared to the dose required to establish infection by the IN route, based on the animal groups inoculated with a low dose of virus and that AERO infected animals transmit infection less efficiently, based on the animal groups inoculated with a high dose of virus. In contrast IN infection, requires a higher dose of virus to infect, but these animals start shedding one day earlier and shed more virus over the duration of virus egress. IN infected animals transmitted virus more efficiently, as all of their contacts were infected. The pattern of viral kinetics of contacts differed from all of the other directly infected groups in terms of the initial viral load and end of the shedding period. Nevertheless, the results for infection kinetics of contact animals, suggest that low dose challenge is more comparable to natural infection, in terms of viral kinetics and that AERO low delivery is more reproducible that IN low.

### Cytokines in nasal swabs

**Figure 2:**
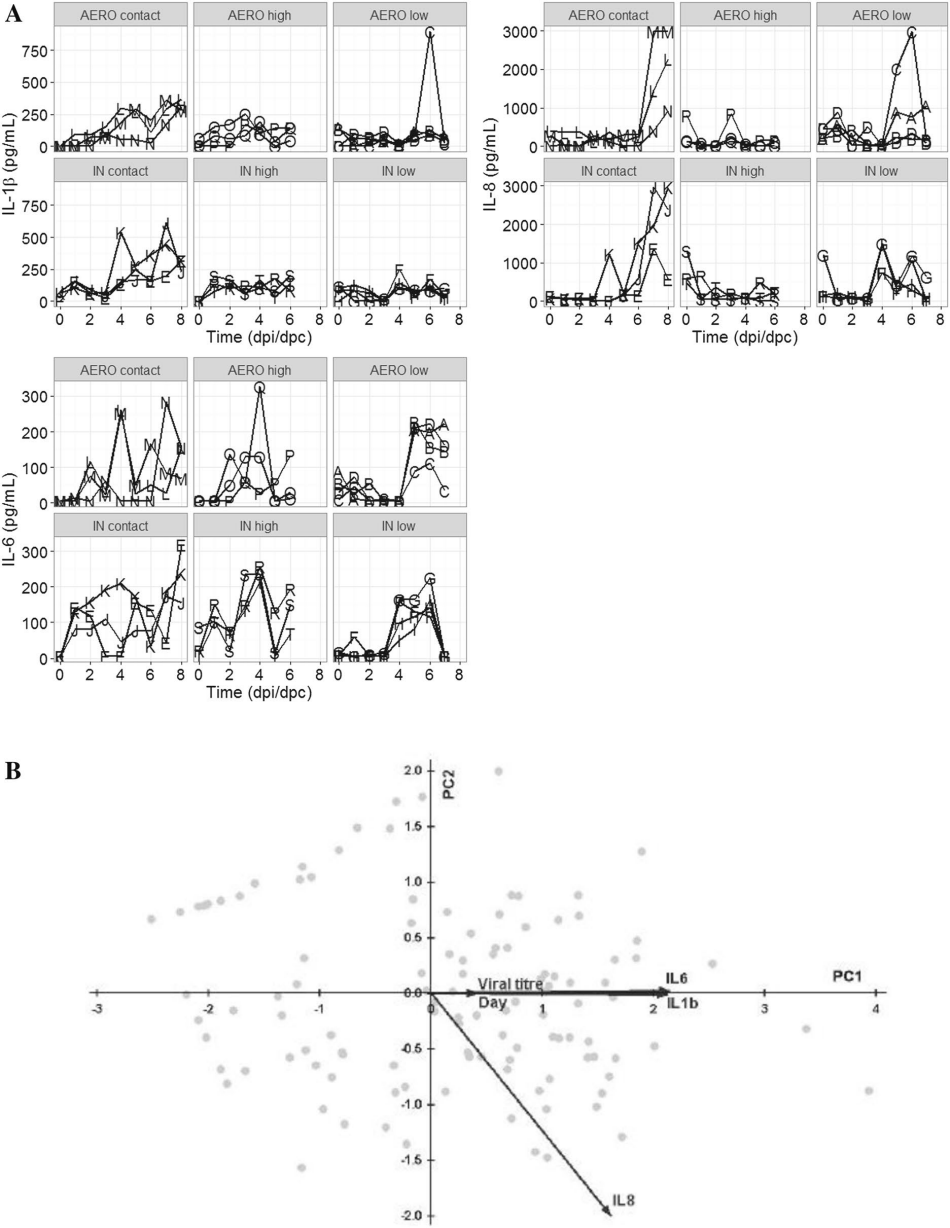
**Cytokines in nasal swabs. A** Concentrations of IL-1β, IL-6 and IL-8 for each infected group. Each line within the graphs represents an individual within the indicated group. **B** Principal component (PC) analysis for IL-6, IL-8 and IL-1β. Scatterplot with vectors showing the direction and strength of the correlation of IL-6, IL-8, IL-1β, virus titre and time along PC1 and PC2.

Cytokines and chemokines are produced early after infection as part of the innate immune response to pathogens and therefore we determined whether the dose and route of infection affects the innate immune response, assessed by analysing cytokine levels in nasal swabs. We measured IFN-α, IFN-γ, TNF-α, IL-1β, IL-4, IL-6, IL-8, IL-10, IL-12, between 0 to 6 dpi or dpc. However only IL-6, IL-8 and IL-1β were used for PCA as they were the only cytokines with greater than half of their measurements above the limit of quantification Figure 2A. We also measured cytokines in serum and BAL fluid but did not find any correlation between the responses in nasal swabs, serum and BAL fluid (Table 2).

**Table 2:** **Serum and BAL fluid A/Sw/Eng/1353/09 specific HAI titres**

Group	Animal	Serum		BALF
		Pre-challenge	14 dpi/16 dpc	14 dpi/16 dpc
AERO low	A	16	32	16
	B	4	64	32
	C	8	64	32
	D	8	32	8
IN low	E	16	64	16
	F	16	16	16
	G	8	128	16
	H	16	32	16
AERO high	I	16	256	64
	J	8	256	64
	K	16	256	64
IN high	L	8	32	16
	M	8	64	16
	N	16	128	16
IN contact	O	16	256	32
	P	16	64	16
	Q	16	64	16
AERO contact	R	8	128	16
	S	16	128	32
	T	8	256	32

HA titres were determined in serum and BAL fluid at day of sacrifice (14 or 16 dpc) using 4 HAI units of A/Sw/Eng/1353/09 virus. Results shown are the mean of all individuals in each of the groups ± standard deviation. Serum from pigs immunized with commercial A(H1N1)pdm09 vaccine Pandemrix (GSK) and challenged with A/England/195/09 (pdmH1N1) virus as previously described [11] was used as a positive control and gave a titer of 2048.

The results of the principal component analysis (PCA) are shown using 2D ordination graphs of the distance between sample units which approximates increasing time (days) and viral titre (Figure 2B). PC1, which explains 78.9% of the variation in the data, is associated with high levels of IL-6 (*p* < 0.001) and IL-1β (*p* < 0.001). IL-8 was associated with both PC1 (*p* < 0.001) and PC2 (*p* < 0.001). PC1 is associated with high virus titre (*p* < 0.001) and increasing time (day) (*p* < 0.001). The contacts were significantly different from the low dose directly challenged pigs by both routes of inoculation (AERO low *p* = 0.02 and IN low *p* = 0.02). However there were no differences between the contacts and the AERO high (*p* = 0.1) or IN high (*p* = 0.1) groups. For low doses there was no significant difference between the routes of inoculation (*p* = 0.8) but the AERO high and IN high groups differed significantly (*p* = 0.006).

These data showed that the cytokine profiles of the naturally infected contacts were more similar to the high dose than low dose directly challenged animals.

### Antibody and cellular immune responses

We next determined whether the dose and route of infection affect the adaptive immune response. HAI titres in serum and BAL fluid are shown in Table 3. The highest HAI titers were detected in the serum of the AERO high (1:256 ± 0) and both contact groups (AERO contact 1:170.7 ± 73.9 and IN contact 1:128.0 ± 110.9). Similarly, the highest HAI titer in the BAL fluid was detected in the AERO high animals (64 ± 0) (Table 3).

**Table 3:** **Cytokine concentrations in serum and BALF of AERO and IN infected pigs**

Group	Animal	IL-10		IL-12p40		IL-8	
		Serum	BALF	Serum	BALF	Serum	BALF
AERO high	I	0.9	ND	216.5	2.4	288.7	260
	J	11.5	ND	184.6	6.1	239.4	141.9
	K	20.3	ND	394.9	3.9	101.9	79.8
IN high	L	ND	ND	490.4	7.5	152.4	523.5
	M	ND	ND	98.6	4.2	173.4	125.5
	N	ND	ND	472.8	2.2	218.8	162.6

Cytokine concentrations (pg/mL) in the serum and BAL fluid at the day of sacrifice of AERO and IN high groups were determined using a commercial Luminex assay.

**Figure 3:**
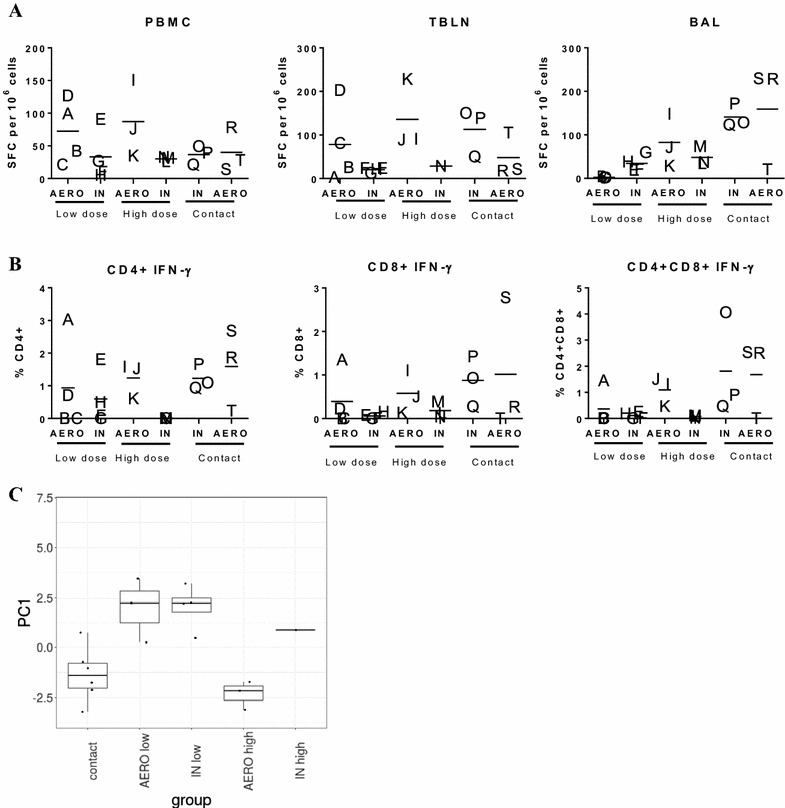
**Cytokine responses in PBMC, BAL and TBLN.** Pigs were inoculated with 1 × 10^4^ EID_50_ (low, circles) or 1 × 10^7^ EID_50_ (high, squares) of A/Sw/Eng/1353/09 virus by AERO (open symbols) or IN (solid symbols) or by contact with the high dose AERO infected pigs (open diamonds) or by contact with the high dose IN infected pigs (solid diamonds) as indicated on the figure. **A** Numbers of IFNγ secreting cells in PBMC, BAL and TBLN at 14 dpi or 16 dpc were determined by ELISPOT. **B** The percentage of IFNγ producing cells in BAL was determined by intracellular cytokine staining and flow cytometry at 14 dpi or 16 dpc. Cytokine expression is presented as a percentage of the total number of cells in CD4+, CD4+CD8+ and CD8+ cells. Each data point represents an individual within the indicated group and the horizontal lines indicates the mean. **C** Box and whisker plots of the scores of PC1 (29.59% explained variance) for immune response in each treatment group. Each boxplot represents the distribution of estimates for pigs in the indicated groups.

We analysed the influenza A virus (1353/09 homologous stimulation) specific T cell responses in PBMC, BAL and TBLN by IFN-γ ELISPOT on 14 dpi and 16dpc (Figure 3A). The AERO high and low groups showed the highest virus specific PBMC responses (mean 87 ± 59 SFC per 10^6^ cells for AERO high, 72 ± 49 for AERO low, compared to 33 ± 39.4 and 30 ± 3.5 for IN high and IN low groups and 40 ± 33.6 and 36.7 ± 14.2 for the AERO and IN contacts). The AERO and IN contacts had the highest numbers of IFN-γ secreting cells in BAL (respectively 159.2 ± 120.3 and 140.9 ± 26.8 SFC per 10^6^ cells); whereas much lower responses were seen in the remaining animals (Figure 3A). In TBLN the AERO high challenge induced the strongest IFN-γ ELISPOT response followed by the IN contacts and AERO low, while responses in both IN groups were very low (Figure 3A).

We performed intracellular staining for IFN-γ following in vitro stimulation with A/Sw/Eng/1353/09. In BAL a similar trend to the IFN-γ ELISPOT was observed with the contacts and AERO high and low directly challenged showing higher IFN-γ response by CD4, CD8 and CD4CD8, compared to IN challenged animals (Figure 3B).

PCA was performed with PC1 explaining 29.6% of the variation in the immune reponses of the different treatment groups and was significantly associated with group (*p* = 0.001) (Figure 3C). There were no significant differences between the contacts and both AERO and IN high dose groups (AERO high *p* = 0.8, IN high *p* = 0.5). In contrast contacts were significantly different from both low dose groups (AERO low *p* = 0.02, IN low *p* = 0.01). There were also no significance differences between the high dose groups (*p* = 0.2) or between the low dose groups (*p* = 1). None of the remaining PCAs were significantly associated with treatment group. Interestingly, when the PC loadings were considered it was apparent that intracellular cytokine production by PBMCs was positively loaded on PCA1, while IFN-γ production by cells in the ELISPOT assay was negatively loaded, indicating that the two techniques measured different immunological parameters (Additional file 3). A possible explanation could be that other cells such as NK cells are detected in the ELISPOT, while ICS detected only CD4 or CD8 T cells. Antibody levels, as measured by HAI in BAL and serum, correlated with IFN-γ secretion by cells in the BAL and with IFN-γ production by CD4^+^CD8^+^ T cells in the TBLN.

In summary, in contrast to the viral kinetics, the antibody and cellular immune responses of the naturally infected contacts were similar to the high dose directly infected animals and significantly different to the low dose animals. Although there was a trend for higher immune responses in the AERO high compared to the IN high animals this did not reach statistical significance.

### Distribution of Evans blue dye in the respiratory tract and lung pathology after AERO and IN delivery

**Figure 4:**
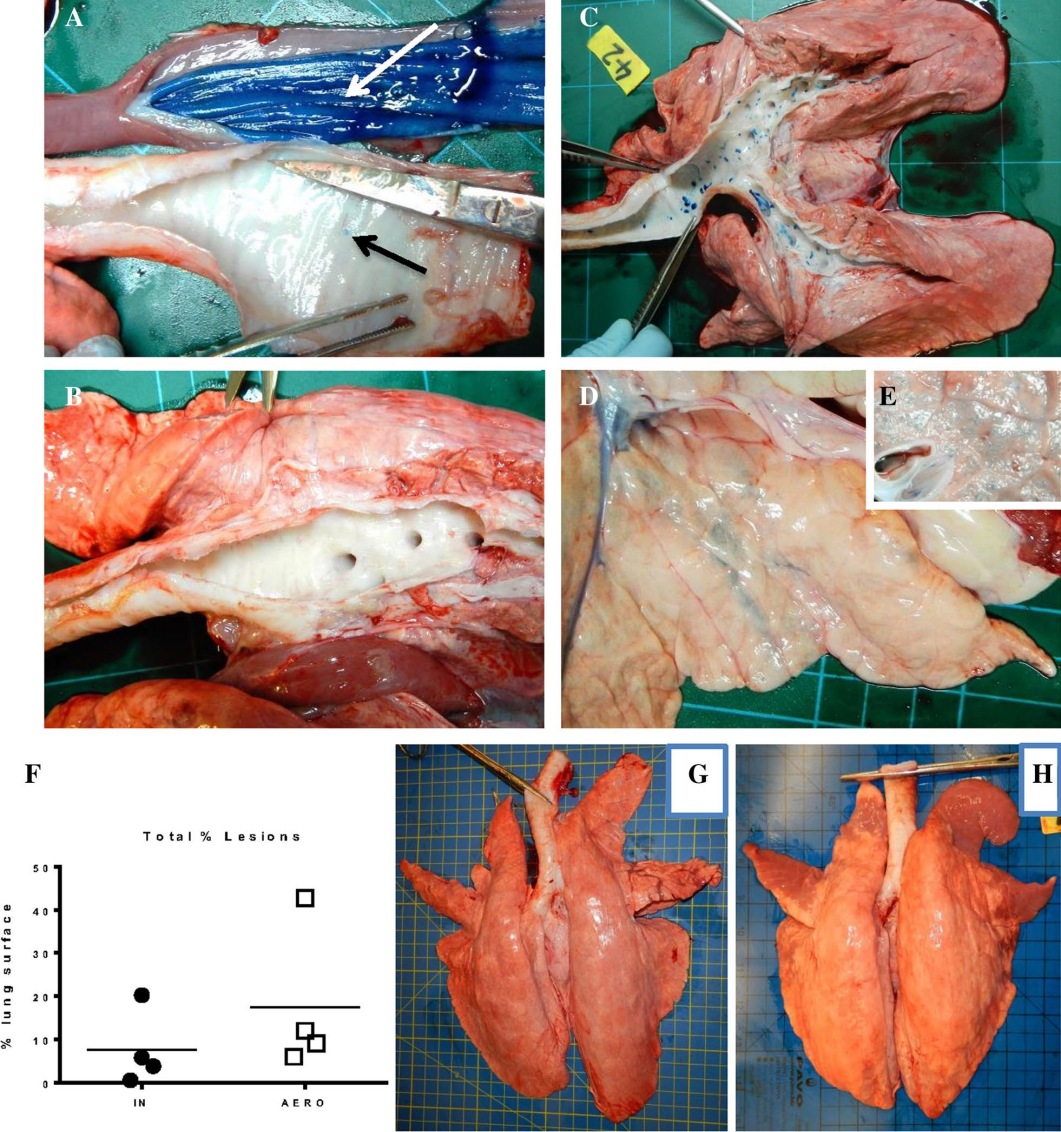
**Distribution of Evans Blue and lesions in lungs.** Groups of six pigs were administered Evans Blue either IN or by AERO. IN delivery of Evans Blue resulted in heavy staining within the alimentary canal (oesophagus, white arrow) and a very small amount in the upper trachea (black arrow) (**A**), no deposit in the lower trachea nor the rest of the respiratory tract or the lung parenchyma (**B**). AERO administration resulted in widespread distribution within the entire respiratory tract (**C**) including the lung parenchyma as observed through the pleura (**D**) or after sectioning the lung (**E**). The percentage of lung surface with influenza-like lesions varied in each animal with a higher score in AERO than IN (**F**). The distribution of lesions showed a small number of lung lobes with lesions in IN (**G**) compared to the majority of lung lobes in AREO (**H**).

To establish the distribution of material after AERO and IN delivery, we examined the respiratory tract after delivery of Evans Blue dye by MAD or aerosol [31]. IN administration using a MAD device resulted in delivery of dye to the URT and the digestive tract. Dye was found within the nasal cavity mucosa, and a very small amount within the larynx and the upper trachea (Figures 4A and B). In addition, considerable quantities of dye could be found in the oral cavity, oropharynx, oesophagus (Figure 4A) and the stomach. This is consistent with the expected deposition profile of droplets of ~105 μm in diameter, while some excess dye also runs down the back of the throat and is swallowed. In these circumstances, any deposition in the lung would be expected to be as a result of aspiration of Evans Blue from the nasopharynx and/or larynx.

After aerosol delivery dye was found within the nasal cavity mucosa, the larynx, trachea and the entire respiratory tree from the large bronchi towards the bronchioles and including the lung parenchyma (alveoli). This is in accord with expectations, as droplets of ~3.8 μm in diameter would efficiently transit through the nasal cavity and into and throughout the LRT. Some deposition would be expected in the nasal cavity given the turbulent airflow through the turbinates (Figures 4C and D). A small amount of dye was found in the oesophagus. This is likely explained by Evans Blue again running down the back of the throat.

Because of the distinct patterns of dye distribution, we compared the lung pathology 4 days after IN and AERO administration of A/Sw/Eng/1353/09 influenza virus. Typical influenza-induced gross pathology was found in lungs from both groups although in the AERO the lesions were affecting different areas of the lung including the more distal parts of the lobes, while the IN, gross lesions were fewer in numbers and localised closer to the big bronchi (Figures 4F–H). The pulmonary lesions consisted of multifocal areas of consolidation, dark in colour and consistent with necrotising bronchiolitis and atelectasis [32].

Taken together these data indicate that IN delivery distributes the dye in the URT and the digestive tract, while AERO results mainly in a wider distribution within the LRT, i.e. respiratory tree, including the lung parenchyma within the proximal and distal parts of all lung lobes. Nevertheless even with IN administration the lung does become infected albeit with a more localised distribution of lesions.

## Discussion

Although aerosol is one of the natural routes of SwIV transmission, very few studies have been performed using aerosol challenge and to date there had been no head-to head comparisons of IN, AERO and natural contact infection. As the pig provides a key component in studying pathogenesis of IAV and assessment of anti-viral therapies, an understanding of the impact of the route of delivery is essential to allow comparison of data generated using different challenge models. Our results indicate that influenza A swine pH1N1 viral kinetics and immune response in naturally infected contact animals differ from those of animals experimentally infected by different doses and routes. In contacts, the kinetics of virus shedding were slow, prolonged and more similar to the low dose directly infected animals. In contrast the cytokine profile in nasal swabs, antibody and cellular immune responses of contacts more closely resemble immune responses in high dose directly inoculated animals.

We also show that successful infection of pigs can be achieved with a much lower dose of virus by AERO than IN administration. Aerosol administration of influenza A virus to human volunteers demonstrates that fewer than ten virions can initiate infection and similar results have been obtained in the ferret model [21, 33, 34]. Additionally, influenza A virus in small droplets has been shown to be 100 times more infectious than influenza virus in large droplets [35]. Although IN challenge required a higher dose for successful infection, once infected, animals produced more virus and were better able to transmit infection, since all of the IN high contacts became infected. In contrast the animals given a high dose of virus by AERO did not transmit efficiently, suggesting that virus replicating in the LRT is more efficiently contained and less likely to be emitted than that in the URT. This is in agreement with transmission studies in ferrets, showing a significant correlation between virus titer in nasal washes and the likelihood of virus transmission, indicating that replication in the URT is the source of virus for both direct contact and airborne transmission [33].

Our results also demonstrate that natural infection is highly efficient in generating strong immune responses. Both innate and adaptive immune responses in the contacts are comparable to the responses in AERO and IN high dose directly challenged animals, yet viral kinetics indicate that the contacts received a low infectious dose. This paradox suggests that natural infection with a low dose is highly efficient in inducing an immune response and the response elicited is similar to that following experimental delivery of a high dose to either to the URT or LRT. We speculate that natural infection targets both the URT and LRT and therefore reaches the greatest number of antigen presenting cells and many lymph nodes, enabling the host to mount a strong and efficient protective immune response. On the other hand from the perspective of the virus, reaching the greatest number of receptor bearing cells gives it the best chance to infect and transmit.

In humans and ferrets aerosols of <5 μm are capable of reaching deep into the LRT and reaching alveolar tissues. Using Evans Blue dye we have shown that MAD delivery of ~105 μm droplets results in deposition in the URT and the digestive tract, while nebuliser-generated aerosol results in a wider distribution within the respiratory tree, including the lung parenchyma within the proximal and distal parts of all lung lobes. At first sight it is surprising that IN and AERO delivery which showed such different patterns of dye delivery, both generated strong immune responses. However this maybe because dye distribution does not represent what happens with infectious SwIV. As Figure 4F shows, pigs administered virus IN or by AERO both showed lung pathology. IN delivery by MAD is a widely used method for experimental pig challenge because of its technical expediency and many studies show that it is reproducible. We hypothesize that with MAD delivery of a high dose of virus sufficient material reaches the LRT to evoke an LRT immune response, although we and others have shown that the most efficient way to reach the LRT and induce an immune response is by aerosol [12, 36–38]. Further work using a larger group of animals will be required to confirm the differences in these readouts between IN, AERO and contact routes of infection.

In summary our data show that the inoculation method used for assessing immunity to SwIV and vaccine efficacy is critical in determining the outcome. High dose IN challenge results in a much higher virus titer in nasal swabs at day 1 compared to contact infected animals and it may be difficult to show protective efficacy, especially of T cell based vaccines, which do not prevent virus entry into cells. While contact infection may be optimal, it requires more animals and is consequently expensive. Low dose aerosol challenge may provide the next best alternative to natural infection. Our comparison of IN, AERO and contact infected animals shows that the route of infection and the dose of infectious virus may affect the outcome in terms of infectivity, viral shedding, immune response and pathology. Consideration of these differences is important for studies of disease pathogenesis and assessment of vaccine protective efficacy.

## Additional files

**Additional file 1.** **Viral titre fits with a segemented linear regression.** Each box represent a challenge group. The dots represent the observed viral titre, the solid line the best fit curve and the grey area best fit curve ± standard error.

**Additional file 2.** **Bland–Altman plot comparing viral titre measured by PCR (REU) and by plaque assay (log10 PFU/mL).** The solid line represents the average difference and the dashed lines the average difference ± 2 standard deviations.

**Additional file 3.** **Principal component analysis for IFNγ ELISPOT, ICS and Ab titers.** PC loadings for PC1 and PC2 (PC2 was not significantly associated with treatment group) showing the amount of variability in PC1 and PC2 that can be explained by each variable. Intracellular cytokine production by PBMCs was positively loaded on PC1, while IFN-γ production by cells in the ELISPOT assay was negatively loaded. BAL samples cluster together and are also negatively loaded. Antibody levels in BAL and serum correlate with IFN-γ secretion by cells in the BAL and with IFN-γ production by CD4^+^CD8^+^ T cells in LN.

## Abbreviations

IAV: influenza A virus
pdm: pandemic
SwIV: swine influenza virus
MAD: mucosal atomisation device
LRT: lower respiratory tract
URT: upper respiratory tract
IN: intra-nasal
AERO: aerosol
HAI: haemagglutination inhibition
MDCK: Madin-Darby canine kidney
EID: egg infectious dose
FBS: foetal bovine serum
BAL: broncho-alveolar lavage
TBLN: trachea-bronchial lymph nodes
Ab: antibody
PFU: plaque forming units
PCA: principal components analysis
MRPP: multi-response permutation procedures
FPF: fine particle fraction
