# Supplementary figures and images for: Distinct immune responses and virus shedding in pigs following aerosol, intra-nasal and contact infection with pandemic swine influenza A virus, A(H1N1)09
Source: Vet Res. 2016 Oct 20;47:103. doi: 10.1186/s13567-016-0390-5 (PMC5073419; doi:10.1186/s13567-016-0390-5)

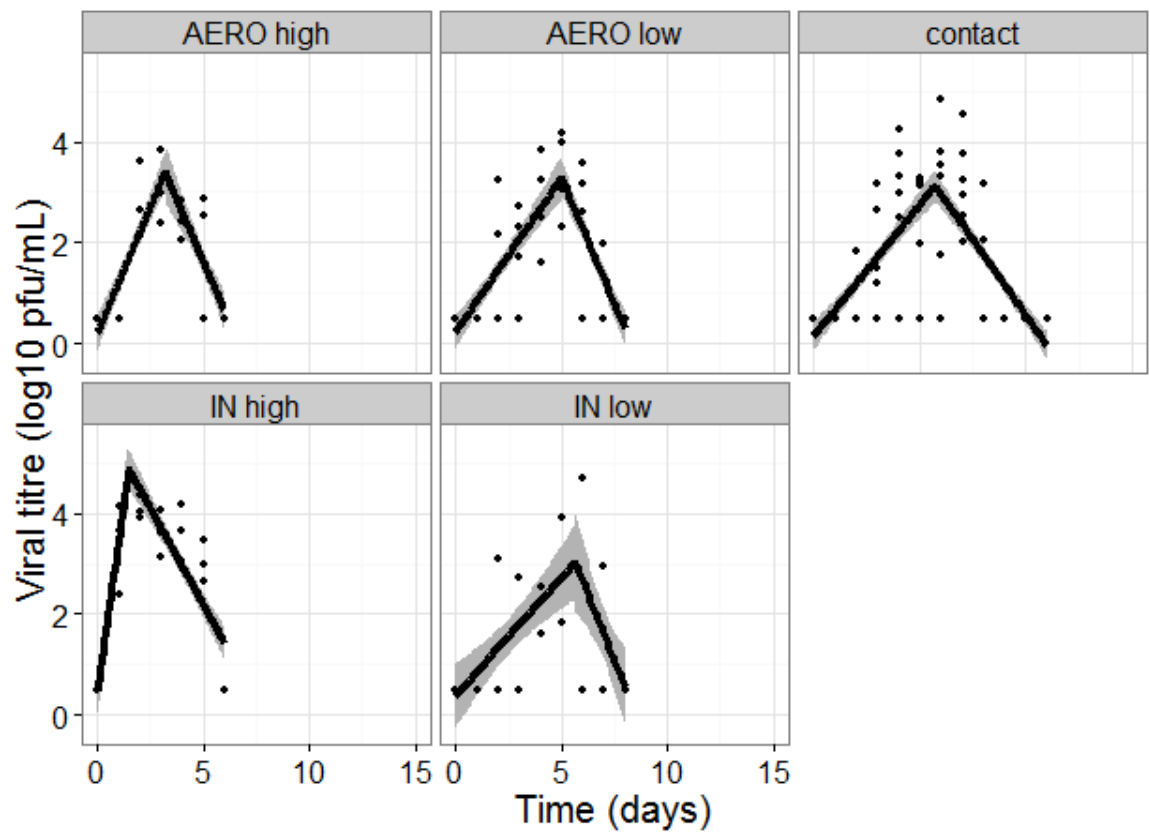

Supplement: Supplementary file 1 — Additional file 1. Viral titre fits with a segemented linear regression. Each box represent a challenge group. The dots represent the observed viral titre, the solid line the best fit curve and the grey area best fit curve ± standard error. [file 13567_2016_390_MOESM1_ESM.pdf]

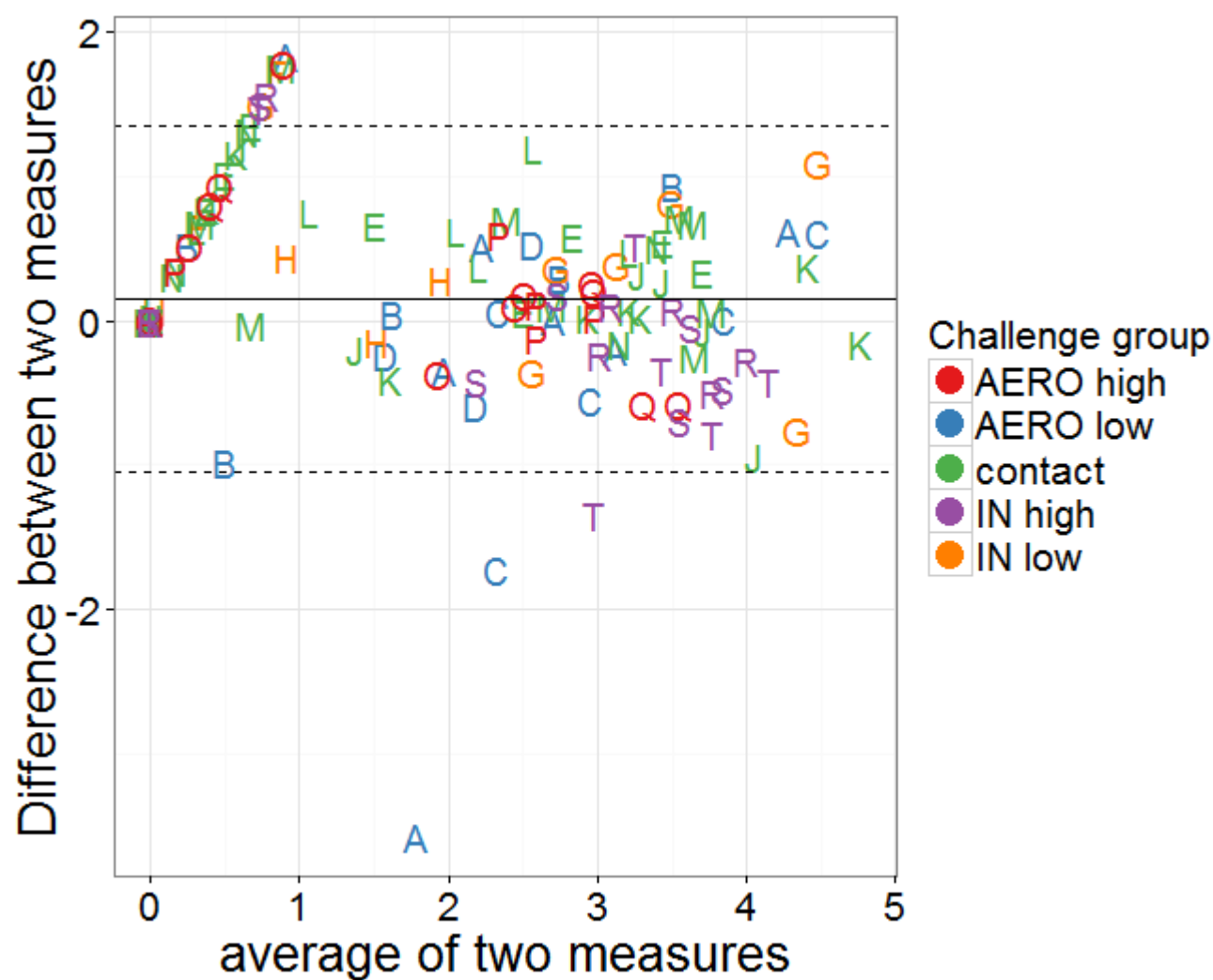

Supplement: Supplementary file 2 — Additional file 2. Bland–Altman plot comparing viral titre measured by PCR (REU) and by plaque assay (log10 PFU/mL). The solid line represents the average difference and the dashed lines the average difference ± 2 standard deviations. [file 13567_2016_390_MOESM2_ESM.pdf]

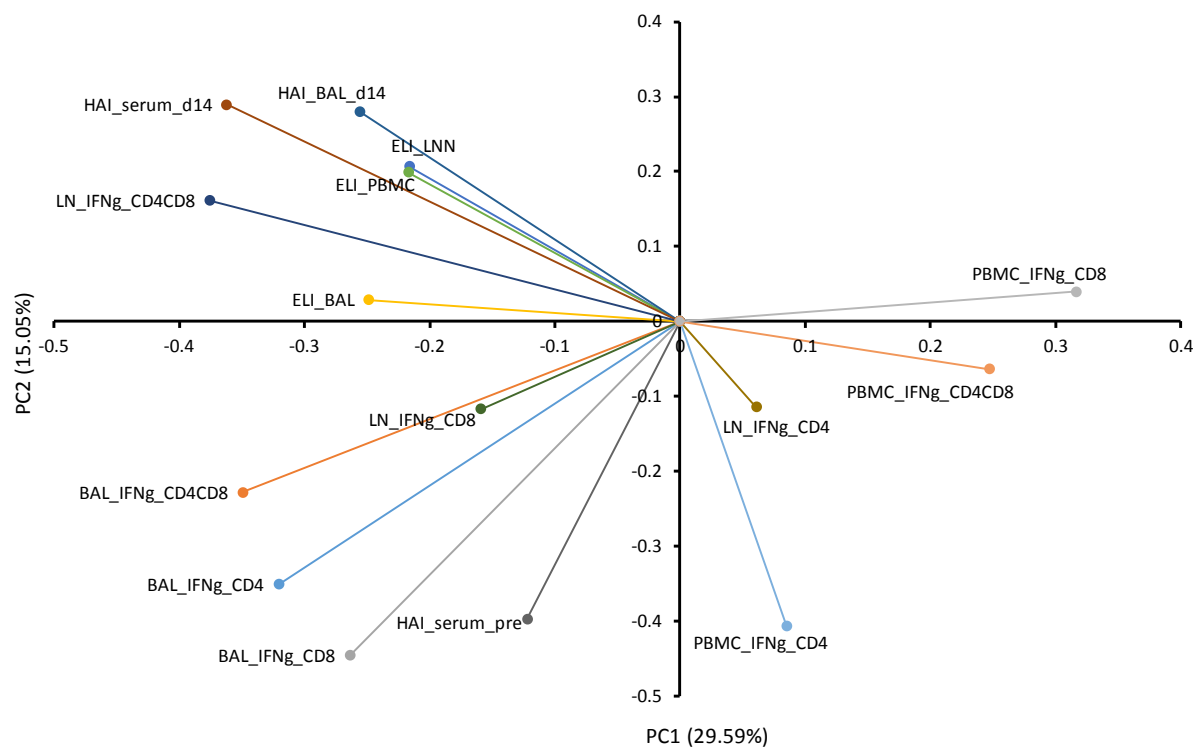

Supplement: Supplementary file 3 — Additional file 3. Principal component analysis for IFNγ ELISPOT, ICS and Ab titers. PC loadings for PC1 and PC2 (PC2 was not significantly associated with treatment group) showing the amount of variability in PC1 and PC2 that can be explained by each variable. Intracellular cytokine production by PBMCs was positively loaded on PC1, while IFN-γ production by cells in the ELISPOT assay was negatively loaded. BAL samples cluster together and are also negatively loaded. Antibody levels in BAL and serum correlate with IFN-γ secretion by cells in the BAL and with IFN-γ production by CD4+CD8+ T cells in LN. [file 13567_2016_390_MOESM3_ESM.pdf]
